# Early epidural lead migration in spinal cord stimulator trials: A case series

**DOI:** 10.1016/j.inpm.2024.100426

**Published:** 2024-07-16

**Authors:** Wendy Han Cong Song, Tim Ting Han Jen, Jill Alison Osborn, Vishal Varshney

**Affiliations:** a Department of Medicine, University of British Columbia, Vancouver, British Columbia, Canada; b Department of Anesthesiology, Pharmacology & Therapeutics, University of British Columbia, Vancouver, British Columbia, Canada; c Department of Anesthesiology, St. Paul's Hospital, Providence Health Care, Vancouver, British Columbia, Canada

**Keywords:** Case series, Complications, Lead migration, Neuromodulation, Spinal cord stimulation, Trial lead placement

## Abstract

**Background:**

Spinal cord stimulation (SCS) devices are routinely trialed to assess pain and functional improvement before permanent lead implantation. Lead migration is a common complication that may cause a loss of therapeutic effect in patients who may otherwise benefit from SCS. The timing of lead migration during the trial period is currently unknown.

**Objectives:**

We hypothesize that significant lead migration may occur early in the SCS trial period, such as postoperative day 1 or 2, which may allow for contact stimulation adjustment to prevent false negative trial results. As such, in this study, we aim to evaluate the incidence and distance of lead migration in early thoracic SCS trial period.

**Methods:**

We performed a case series of 27 patients ≥19 years of age who received differential target multiplexed thoracic SCS trials for chronic neuropathic pain from July 1, 2020 to July 1, 2023. Patients with a neuropathic pain diagnosis failing medical treatment, without structural pathology limiting epidural access, and with psychiatric clearance for suitability are eligible for SCS trials at our center. Pre- and post-flexion radiographs taken immediately after implantation and on postoperative day 1 or 2 were examined to assess the distance of lead migration. Clinically significant lead migration was pre-defined as ≥ 10 mm.

**Results:**

The mean (SD) distances of epidural lead migration on postoperative day 1 or 2 were 18.2 (12.9) mm and 19.1 (13.3) mm for the cephalic and caudal leads, respectively. All migrations were caudad except for one trial. Clinically significant lead migration occurred in 20/27 (74 %) patients.

**Conclusion:**

Clinically significant epidural lead migration occurs in the early SCS trial period.

## 1 Introduction

Spinal cord stimulation (SCS) use in the United States has steadily increased over the past decade, with approximately 1 million Medicare-approved SCS placements from 2000 to 2019 [1,2]. Clinical indications of SCS include focal chronic pain syndromes refractory to conventional therapies, such as chronic pain after spinal surgery, complex regional pain syndrome, and painful diabetic neuropathies [3]. A trial of SCS is routinely performed before permanent implantation to ascertain therapeutic efficacy in pain relief and functional improvement [3,4]. SCS trial leads can be placed percutaneously and are attached to an external pulse generator for an average duration of 5–7 days [5].

Electrode placement at specific anatomical locations is crucial for accurate stimulation with the advent of anatomical-based spinal cord stimulation programming. Differential targeted multiplexed (DTM) programming is one such modality that requires stimulator electrodes to span the T8-T10 spinal levels in order to deliver optimal therapy for management of chronic low back and/or leg pain [6]. This is a paresthesia-free form of spinal cord stimulation requiring specific anatomical electrode placement at these levels based on proposed mechanism of action of glial cell modulation. This differs from the previously commonly used tonic stimulation, which is typically paresthesia-based.

Unfortunately, trial lead migration is a common issue that may lead to loss of efficacy and false negative trial results. With the advent of paresthesia-free, high-frequency stimulation, radiographically significant lead migration can be detected using serial radiographic imaging, often performed at the end of the trial period [[7], [8], [9], [10]]. Some SCS programs stimulate specific contacts of the trial leads, and lead migration can become clinically significant when stimulated contacts migrate away from the targeted anatomical area [11]. Published rates of clinically significant migration of at least one trial lead vary between 0.7 % and 94 % [9,10,12]. Risk factors of lead migration include increased BMI and certain anchoring techniques [7,10,13,14]. However, it is currently unknown whether lead migration occurs early or late in the trial period. It is conceivable that if lead migration occurs early in the SCS trial period and the degree of migration is quantified, contact stimulation may be adjusted accordingly to prevent false negative trial results and to better inform clinicians regarding the precise location of stimulation that confer the maximal therapeutic effect.

As such, the study objective is to perform a retrospective case series of patients who underwent Medtronic SCS trials at St. Paul's Hospital, Vancouver, Canada to quantify the incidence and distance of clinically significant lead migration in the early SCS trial period and evaluate its impact on trial success.

## 2 Methods

The study received Research Ethics Board approval (The University of British Columbia Providence Health Care Research Ethics Board; H23-02172; August 14, 2023). The protocol and statistical analysis plan were preregistered at Open Science Framework (10.17605/OSF.IO/XGWFP; October 3, 2023) before data analysis. This manuscript adheres to the Preferred Reporting of Case Series in Surgery (PROCESS) guideline [15].

The study is a single-centered retrospective case series. Inclusion criteria are patients aged 19 years or above who received differential target multiplexed (DTM) thoracic SCS trials with Medtronic Vectris™ SureScan™ MRI Subcompact 977A2 Lead Series electrodes (Medtronic PLC, Minneapolis, MN, USA) for chronic neuropathic pain from July 1, 2020 to July 1, 2023 at the Interventional Pain Clinic of St. Paul's Hospital, Vancouver. St. Paul's Hospital is a Canadian, tertiary-care, academic institution with over 100 SCS trials and implants yearly, and SCS trials are performed by staff clinicians without trainees over this time period. Study exclusion criteria were trials using other devices and programming, cervical or lumbar lead placements, and missing or incomplete radiographs on postoperative day 1 or 2 after trial lead placement.

Our institutional eligibility criteria for SCS trials are patients with a chronic focal neuropathic pain diagnosis failing medical treatment (examples delineated in Table 2), without structural pathology that limits epidural access, and with psychiatric clearance for suitability. SCS leads are placed by obtaining epidural access at the L1-2 or L2-3 vertebral levels, with needle skin entry point at the immediately inferior pedicle of the vertebral body. Epidural access is obtained at these levels using a 14G Tuohy needle and a loss-of-resistance to saline technique. To facilitate DTM programming, staff clinicians insert two leads via the 14G Tuohy needle, and these are placed with electrodes spanning from the superior endplate of the T8 vertebral body to mid-T10 vertebral body. Immediately after lead placement, a “pre-flexion” radiograph is taken with superior end plate of T8 squared off. Leads are secured using 2-0 silk suture to skin, Mastisol liquid skin adhesive (Eloquest Healthcare, Ferndale, MI, USA), Biopatch dressing (Ethicon, Raritan, NJ, USA), and Epiguard dressing (Dyna Medical, London, ON, Canada). Patients then perform forward flexion of their thoracolumbar spine repeatedly. Thereafter, a “post-flexion” radiograph is taken with the superior end plate of T8 squared off. Of note, the process of taking post-flexion radiographs began May 2022. No intraoperative paresthesia-mapping is performed, with initial patient programming based on the electrode position as seen on the post-flexion radiograph being completed while the patient is in the post-operative holding area. Radiographs are taken again in the ‘early trial period’, defined as postoperative day 1 or 2, and, if lead migration is detected, adjustments to the programming are done in our outpatient clinic to select and program electrode contacts located at the T8-10 anatomical levels. Clinic nursing staff follow patients throughout the trial duration to assess therapeutic effect and to detect possible adverse events.

The primary outcomes are the incidence and distance of lead migration in the early trial period, as measured by the distance between lead locations of the pre-flexion radiograph with the early trial period radiograph. Measurement was performed using the image ruler tool of the Philips IntelliSpace Picture Archiving and Communication System 4.4 Enterprise (Philips Healthcare, Andover, MA, USA). A magnification factor is applied based on the length of one Medtronic 977A2 series electrode contact (7 mm) divided by the measured lead length on the radiograph [7,10]. Secondary outcomes are the incidence and distance of lead migration after thoracolumbar flexion immediately following trial lead placement.

Data were extracted from electronic medical records of Clinical & Systems Transformation Cerner (Oracle Cerner, Kansas City, MO, USA). Cohort characteristics were age, sex, height, weight, BMI, underlying pathology, method of lead fixation, epidural access level, trial duration, and trial success. Trial success was defined as improvement of ≥5 points in ≥3 categories of the PROMIS-29 survey or ≥50 % reduction in pain intensity on an 11-point visual analogue scale (VAS).

### 2.1 Statistical methods

Patient demographics were summarized descriptively, with continuous variables reported as mean (standard deviation [SD]) if normally distributed or median (inter-quartile range [IQR]) if not normally distributed, and categorical variables were reported as number and frequency (%). For the primary outcome variable of lead migration distance, normality was assessed using Shapiro-Wilk's test and visual inspection of Q-Q plot given the limited sample size. Skewness, kurtosis, and histogram of primary outcome data were assessed. Statistical significance was defined as p < 0.05. Clinically significant lead migration distance was pre-defined as distance greater than or equal to 50 % of a full vertebral level, correlating with 10 mm assuming average vertebral body and disc height of 24 mm in the mid- and lower thoracic spine [9,10]. Data were analyzed using R (version 4.0.3; The R Foundation of Statistical Computing, Vienna, Austria). The sample size was determined through convenience sampling of all eligible patients who underwent SCS trials during the study period.

## 3 Results

During the study period, 31 patients receiving Medtronic SCS trial lead placements were assessed for eligibility. Four patients were excluded for receiving permanent implantations with surgical paddle leads without trial, and 27 patients were included in the study. Patient demographic details are summarized in Table 1. The mean (SD) age was 56.7 (16.3) years, with 56 % (15/27) female. The most common indication for SCS trial was chronic pain after spinal surgery (20/27; 74 %), followed by non-surgical low back or leg pain (5/27; 19 %). Trial duration ranged from 8 to 21 days, with a median [IQR] duration of 11 [10, 13.5] days. Three of 27 trials (11 %) were unsuccessful, of which one patient had a complication of query cerebrospinal fluid leak leading to a prolonged trial period of 21 days.

**Table 1:** Patient demographics (n = 27).

Age (SD) years	56.7 (16.3)
Sex n (%)	
Male	12 (44)
Female	15 (56)
Height [IQR] cm	175 [168, 181]
Weight (SD) kg	78.3 (17.2)
BMI (SD)	26.0 (5.1)
Indication for SCS Trial n (%)	
Chronic pain after spinal surgery	20 (74)
Non-surgical low back/leg pain	5 (19)
Structural deformity	1 (4)
Diabetic peripheral neuropathy	1 (4)
Method of Lead Fixation n (%)	
Silk suture, Biopatch, Epiguard	27 (100)
adhesive, and Mepore dressing	
Epidural Access Level n (%)	
T11-T12	1 (4)
T12-L1	1 (4)
L1-2	11 (41)
L2-3	3 (11)
L3-4	2 (7)
Not specified	9 (33)
Trial Duration [IQR] days	11 [10, 13.5]
Trial Success n (%)	
Successful	24 (89)
Unsuccessful	3 (11)

**Table 2:** Clinically significant lead migration.

	Clinically Significant Migration	No Clinically Significant Migration
Immediate Post-Flexion (n = 21)		
Cephalad Lead n (%)	11 (52)	10 (48)
Caudal Lead n (%)	8 (38)	13 (62)
Both Leads n (%)	8 (38)	13 (62)
Early Trial Period (n = 27)		
Cephalad Lead n (%)	20 (74)	7 (26)
Caudal Lead n (%)	20 (74)	7 (26)
Both Leads n (%)	20 (74)	7 (26)

**Fig. 1:**
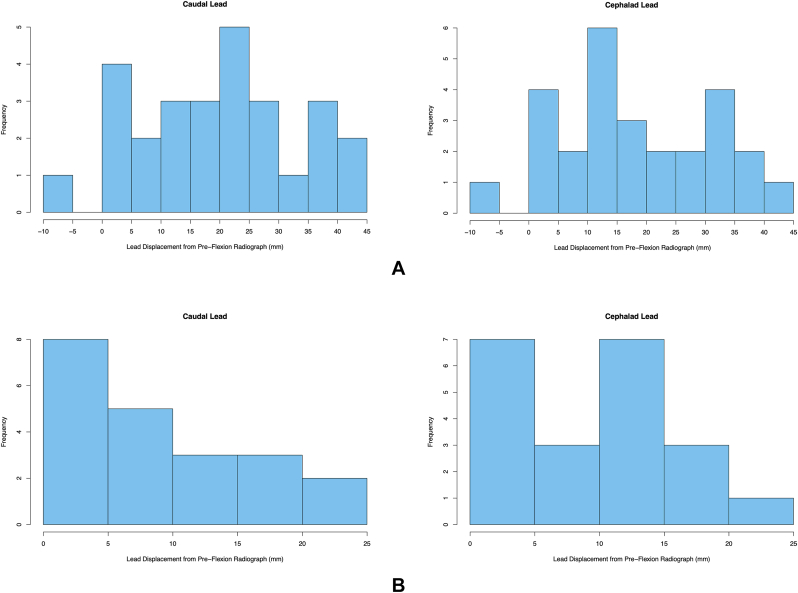
Histogram of Distance of Epidural Cephalad and Caudal Lead Migration. (a) Early trial period of postoperative day 1 or 2. (b) Immediately post-flexion.

For the primary outcome of lead migration in the early trial period, there was clinically significant migration of both leads in 20/27 (74 %) of patients (Table 2). Shapiro-Wilk's test (cephalic lead p = 0.78; caudal lead p = 0.70) and visual inspection of Q-Q plots suggest normal distribution for migration distance. The mean (SD) distance of migration were 18.2 (12.9) mm and 19.1 (13.3) mm for the cephalic and caudal leads, respectively (Table 3). Histograms (Fig. 1), skewness (0.00 and −0.113), and kurtosis (2.16 and 2.19) of the cephalic and caudal leads suggest symmetrical and slightly platykurtic distributions. All migrations were caudad, except for one successful trial with cephalad migration.

**Table 3:** Mean lead displacement from pre-flexion radiograph.

Immediate Post-Flexion (n = 21)	
Cephalad Lead (SD) mm	9.0 (6.5)
Caudal Lead (SD) mm	8.8 (7.5)
Early Trial Period (n = 27)	
Cephalad Lead (SD) mm	18.2 (12.9)
Caudal Lead (SD) mm	19.1 (13.3)

Immediate post-flexion radiographs were taken for the 21 patients who received SCS trials after May 2022. With repeated thoracolumbar spine flexion, there were clinically significant migrations of one (3/21; 14 %) or both leads (8/21; 38 %) (Table 2). Shapiro-Wilk's test (cephalic lead p = 0.36; caudal lead p = 0.08) and visual inspection of Q-Q plots suggest normal distribution for migration distance. The mean (SD) distance of migration were 9.0 (6.5) mm and 8.8 (7.5) mm for cephalic and caudal leads, respectively (Table 3). Histograms (Fig. 1), skewness (0.27 and 0.48), and kurtosis (2.33 and 2.05) of cephalic and caudal leads suggest a mild rightward skew and a slight platykurtic distribution. All migrations were caudad. All patients with clinically significant migration in the post-flexion radiograph had clinically significant migration on the early trial period radiograph.

Of 20 patients who had clinically significant lead migration in the early trial period, 19 (95 %) had successful trials, while 5/7 (71 %) patients without clinically significant lead migration had successful trials (Table 4). All 20 patients who experienced clinically significant lead migration required adjustments to programming in the initial POD1 or POD2 period. Patients that did not experience clinically significant lead migration did not require any programming adjustments on POD1 or POD2.

**Table 4:** Trial lead migration in the early trial period versus trial success.

Trial Lead Migration	Trial Successful	Trial Unsuccessful
Clinically Significant	19	1
Non-Clinically Significant	5	2

## 4 Discussion

Our study demonstrated that for patients undergoing thoracic SCS trials, clinically significant epidural lead migration occurred within the early trial period of postoperative day 1 or 2. In our study, epidural leads migrated frequently (74 %) with an average distance of 18.2–19.1 mm, which is slightly less than one vertebral body and disc height of the thoracic spine. We found that half of the clinically significant lead migrations occurred immediately after lead insertion with repeated thoracolumbar spine flexions, while the other half occurred by the early trial period. Furthermore, the trial lead migration distance doubled by the early trial period when comparing with the migration distance immediately post-flexion. Despite this, our institutional trial success rate remained high at 89 %.

Our study results were comparable to those in existing literature. Jenkinson et al. examined a series of 35 patients undergoing SCS trials and assessed lead migration at the end of the trial period [9]. Using the same definition, Jenkinson et al. demonstrated that 77.1 %–79.4 % of trial leads had clinically significant migrations, with a mean (SD) distance of 1.17 (0.88) vertebral levels. Although 94 % of patients had clinically significant migration of at least one lead, the trial success rate was 88.5 % [9]. Similarly, Mullins et al. evaluated a cohort of 100 patients undergoing SCS trials using devices from various manufacturers, with radiographs taken immediately after lead insertion and at the end of the trial just before lead removal [10]. Clinically significant migration of at least one lead occurred in 62 % of patients, with a mean (SD) distance of 12.5 (18.2) mm, and the trial success rate was 78 % [10]. Finally, Mekhail et al. in 2011 described a retrospective review of 707 SCS trials where trial lead migration occurred in 5 patients (0.7 %), although the nature and extent of migration was not further described [12].

Our study contributed to existing literature by establishing and narrowing the timing of the epidural lead migration to the early trial period of postoperative day 1 or 2. It was previously unknown whether lead migration occurred early or late in the trial period as previous studies utilized radiographs taken only at the end of the trial [[7], [8], [9], [10]]. Early detection of lead migration allows contact stimulation to be adjusted accordingly to prevent false negative trial results. In this study, contact stimulation was adjusted based on lead migration distance using proprietary methods as per the Medtronic DTM protocol, and trial success rate remained high despite a high rate of clinically significant trial lead migration. Additionally, our study confirmed previous findings [9,10] that the average distance of trial lead migration was approximately half to one thoracic vertebral body and disc height. The full DTM trialing protocol requires selecting electrode cathode-anode configurations between the mid-T8 level and the T9-10 disc space levels. Clinicians may thus consider inserting trial leads approximately half a vertebral body cephalic to the targeted area to account for anticipated migration, which would allow patients to trial all components of the DTM trialing protocol, even accounting for migration similar to what was seen in our study.

Our study has several strengths. First, we pre-registered our protocol before data extraction and analysis to improve transparency. Second, we utilized a standardized method of lead insertion with a specified target from the T8 superior endplate to mid-T10 vertebral body. This is important as the distance of lead migration likely depends on the location of lead placement due to varying degrees of spine movements at each vertebral level [16]. Third, our study assessed the possibility of detecting lead migration immediately after lead placement, after patients perform forward flexion of their thoracolumbar spines.

There are some limitations to our study. First, sample size was small and based on convenience sampling technique, which precluded us from examining the association between lead migration and trial success further. Second, there was conflict of interest of two authors with the manufacturer of the device utilized in our study. Nevertheless, we attempted to address this potential bias by excluding these two authors from statistical planning or analysis and by pre-registering our protocol. Third, only the 21 patients who received SCS trials after May 2022 performed repeated thoracolumbar flexion after lead insertion and received post-flexion radiographs. This may have introduced a systemic difference in lead migration and device programming before and after the change. Finally, there may be a systemic difference between posterior-anterior and anterior-posterior imaging due to parallax error [17], and we attempted to account for this using standardized image measuring methods described.

## 5 Conclusion

In conclusion, our study demonstrated that clinically significant epidural lead migration occurs in the early SCS trial period. Future studies may consider formally assessing the association between trial lead migration, trial failure, and therapeutic benefits. Future studies may also consider examining the clinical impact of inserting trial leads half a vertebral body cephalic to the targeted area to evaluate a more comprehensive trial of programming.

## Funding

This research did not receive any specific grant from funding agencies in the public, commercial, or not-for-profit sectors.

## Declaration of competing interest

The authors declare the following financial interests/personal relationships which may be considered as potential competing interests:

Jill Osborn reports a relationship with Medtronic that includes: board membership and speaking and lecture fees.

Jill Osborn reports a relationship with Abbott that includes: board membership, speaking and lecture fees, and travel reimbursement.

Jill Osborn reports a relationship with Edwards Medical that includes: board membership, speaking and lecture fees, and travel reimbursement.

Jill Osborn reports a relationship with Canadian Neuromodulation Society that includes: board membership.

Vishal Varshney reports a relationship with Medtronic that includes: speaking and lecture fees.

Vishal Varshney reports a relationship with Abbott that includes: speaking and lecture fees.

Vishal Varshney reports a relationship with Canadian Neuromodulation Society that includes: board membership.

If there are other authors, they declare that they have no known competing financial interests or personal relationships that could have appeared to influence the work reported in this paper.

## References

[1] Chow C., Rosenquist R. (2023). Trends in spinal cord stimulation utilization: change, growth and implications for the future. Reg Anesth Pain Med.

[2] Romaniuk M., Mahdi G., Singh R., Haglin J., Brown N.J., Gottfried O. (2022). Recent trends in Medicare utilization and reimbursement for spinal cord stimulators: 2000–2019. World Neurosurgery.

[3] Hong A., Varshney V., Hare G.M.T., Mazer C.D. (2020). Spinal cord stimulation: a nonopioid alternative for chronic pain management. CMAJ (Can Med Assoc J).

[4] NICE (2008).

[5] Shanthanna H., Eldabe S., Provenzano D.A. (2023). Evidence-based consensus guidelines on patient selection and trial stimulation for spinal cord stimulation therapy for chronic non-cancer pain. Reg Anesth Pain Med.

[6] Fishman M., Cordner H., Justiz R. (2021). Twelve-Month results from multicenter, open-label, randomized controlled clinical trial comparing differential target multiplexed spinal cord stimulation and traditional spinal cord stimulation in subjects with chronic intractable back pain and leg pain. Pain Pract.

[7] Osborne M.D., Ghazi S.M., Palmer S.C., Boone K.M., Sletten C.D., Nottmeier E.W. (2011). Spinal cord stimulator--trial lead migration study. Pain Med.

[8] Kim C.H., Green A.W., Rodgers D.E., Issa M.A., Ata M.A. (2013). Importance of axial migration of spinal cord stimulation trial leads with position. Pain Physician.

[9] Jenkinson R.H., Wendahl A., Zhang Y., Sindt J.E. (2022). Migration of epidural leads during spinal cord stimulator trials. J Pain Res.

[10] Mullins C.F., Royds J., Al-Kaisy A. (2023). Radiographic lead migration in percutaneous spinal cord stimulator trials. Reg Anesth Pain Med.

[11] Jenkinson R.H., Wendahl A., Zhang Y., Sindt J.E. (2023). Creating realistic definitions of clinically significant radiographic lead migration - a response to “migration of epidural leads during spinal cord stimulator trials”. J Pain Res.

[12] Mekhail N.A., Mathews M., Nageeb F., Guirguis M., Mekhail M.N., Cheng J. (2011). Retrospective review of 707 cases of spinal cord stimulation: indications and complications. Pain Pract.

[13] Dombovy-Johnson M.L., D'Souza R.S., Ha C.T., Hagedorn J.M. (2022). Incidence and risk factors for spinal cord stimulator lead migration with or without loss of efficacy: a retrospective review of 91 consecutive thoracic lead implants. Neuromodulation.

[14] Shaparin N., Gritsenko K., Agrawal P. (2019). A retrospective case series of a novel spinal cord stimulator trial technique with less displacement and migration of the trial leads. Pain Res Manag.

[15] Agha R.A., Sohrabi C., Mathew G. (2020). The PROCESS 2020 guideline: updating consensus preferred reporting of CasESeries in surgery (PROCESS) guidelines. Int J Surg.

[16] Tekmyster G., Jonely H., Lee D.W. (2023). Physical therapy considerations and recommendations for patients following spinal cord stimulator implant surgery. Neuromodulation.

[17] Ellingson A.M., Boelter K., Sembrano J.N., Takahashi T., Polly D.W. (2022). Intraoperative stitched fluoroscopic images: effect of parallax on angular measurements of the spine. Spine J.

